# Strain-rate-dependent plasticity of Ta-Cu nanocomposites for therapeutic implants

**DOI:** 10.1038/s41598-023-43126-6

**Published:** 2023-09-22

**Authors:** Arash Kardani, Abbas Montazeri, Herbert M. Urbassek

**Affiliations:** 1 Computational Nanomaterials Lab (CNL), Faculty of Materials Science and Engineering, K. N. Toosi University of Technology, Tehran, Iran; 2 Physics Department and Research Center OPTIMAS, University Kaiserslautern-Landau, Erwin-Schrödinger-Straße, 67663 Kaiserslautern, Germany

**Keywords:** Nanoscale materials, Theory and computation

## Abstract

Recently, Ta/Cu nanocomposites have been widely used in therapeutic medical devices due to their excellent bioactivity and biocompatibility, antimicrobial property, and outstanding corrosion and wear resistance. Since mechanical yielding and any other deformation in the patient's body during treatment are unacceptable in medicine, the characterization of the mechanical behavior of these nanomaterials is of great importance. We focus on the microstructural evolution of Ta/Cu nanocomposite samples under uniaxial tensile loading conditions at different strain rates using a series of molecular dynamics simulations and compare to the reference case of pure Ta. The results show that the increase in dislocation density at lower strain rates leads to the significant weakening of the mechanical properties. The strain rate-dependent plastic deformation mechanism of the samples can be divided into three main categories: phase transitions at the extreme strain rates, dislocation slip/twinning at lower strain rates for coarse-grained samples, and grain-boundary based activities for the finer-grained samples. Finally, we demonstrate that the load transfer from the Ta matrix to the Cu nanoparticles via the interfacial region can significantly affect the plastic deformation of the matrix in all nanocomposite samples. These results will prove useful for the design of therapeutic implants based on Ta/Cu nanocomposites.

## Introduction

Ta/Cu nanocomposites (NCs) have been widely used in dental implants due to their excellent biocompatibility, antibacterial properties, blood compatibility, and enhanced bone ingrowth^1–5^. Despite their excellent biological properties, the application of such implants for clinical purposes is often accompanied by several challenges due to their mechanical properties. Based on the previous biomechanical studies, two critical issues need to be considered in the design of Ta-based dental implants. First, the mechanical properties of these nanomaterials, including Young's modulus, yield strength, and ultimate tensile strength (UTS), should be similar to those of the adjacent jawbone according to Wolff's law^6,7^. The second is implant loosening, which can occur in non-routine activities such as trauma from an accident. Therefore, it is crucial to thoroughly investigate the mechanical characteristics of implanted materials. Previous studies have shown that the nature of the Ta/Cu interface, grain size, density of grain boundaries (GBs), stacking faults (SFs), and temperature are the most important factors affecting the mechanical behavior of these polycrystalline nanomaterials^8–13^. In this context, the mechanisms governing the plastic deformation of Ta and Ta/Cu NC samples have recently been analyzed in detail^14,15^.

Apart from the aforementioned factors, a literature review reveals that strain rate affects the mechanical features of medical implants at four stages: implant design, production, post-surgery, and during service within the patient's body^16–19^. In this regard, Figueiredo et al.^20^ studied the effects of strain rate on the mechanical behavior of chitosan-based dense materials. They found that the flow stress of such materials is highly sensitive to changes in strain rate. However, they emphasized that high strain rates are not expected during the early stages of medical treatment; instead, they often occur after treatment. On the other hand, the geometrical features of implants, such as pore size and distribution, surface roughness, and number and configuration of implant screws, are design requirements that can prevent crack formation at high strain rate conditions during the production process^21–25^. Additionally, low strain rates can also pose challenges for therapeutic medical implants. It has been shown that stress at low strain rates can facilitate the degradation rate of biodegradable implants within the patient's body^26^. Faraji et al^27^ proposed a new experimental method to evaluate stress corrosion cracking of biodegradable Mg implant alloys under slow strain rates for better control of the degradation degree. Strain rate not only affects the implants themselves but also has a direct impact on adjacent bones. Biological research demonstrates that different strain rates during walking, running, and other activities can directly alter the bone remodeling rate around implants^19,28,29^. It is worth mentioning that strain rate also influences the mechanical behavior of polycrystalline Ta-based implants^30–32^. Zhou et al.^33^ experimentally analyzed the effect of strain rate on the Ta/Cu interface region. It was inferred that increasing the strain rate would promote the interfacial activity, thus facilitating the dislocation transfer between the two phases. Using laser-induced shock loading tests, Florando et al.^34^ investigated the effect of this parameter on the plastic deformation mechanisms of pure Ta. They found that at higher strain rates, plastic deformation occurs by twinning, which was attributed to the lack of active dislocation slipping at these loading conditions. However, when BCC-structured metals undergo extreme deformation rates, dislocation slip or twinning cannot occur to release the induced stress^35–37^. For Ta-based samples, it has been shown that under such severe plastic deformation processes, a phase transition from the BCC crystal structure to the FCC or HCP crystal structure occurs by breaking the bonds of the BCC atoms^38^. It is worth noting that the mentioned process is not a unique phenomenon for BCC metals and similar trends have also been reported in other crystalline structures at extreme deformation regimes^39–41^.

A review of the current literature shows that while the strain rate-dependent mechanical properties of Ta-based polycrystals have been extensively studied^42–45^, their plastic deformation mechanisms at various strain rates have rarely been investigated at the atomic scale. It is worth noting that the mechanisms governing the induced plasticity in these nanomaterials involve only a small number of atoms or atomic layers, which cannot be easily investigated by experimental-based techniques. In this regard, molecular dynamics (MD) simulation, a powerful tool for probing atomic-scale phenomena, can be implemented to explore the strain rate-dependent microstructural evolution occurring in these nanostructured materials^46,47^. Using MD simulations, Hahn et al.^48^ explored the relationship between strain rate and plastic deformation mechanisms of polycrystalline Ta samples. They observed that at the conventional strain rate of 10^7^–10^10^ s^-1^, dislocation slip is responsible for their deformation. It was also found that at very high strain rates (e.g., 10^13^ s^-1^), grain boundary (GB) decohesion leads to the failure of the aforementioned samples. Using the same approach, Pan et al.^49^ investigated the strain rate dependence of the mechanical features of the fine-grained Ta samples. Among them, the flow stress showed the highest correlation with strain rate, which would be manifested in the change of plastic deformation mechanisms at different strain rates. In another study, Li et al.^50^ performed MD simulations on single crystalline Ta structures to understand the effect of strain rate on the elastic properties of these nanostructures. They also detected the discussed BCC-to-FCC phase transition in the mentioned Ta samples at the strain rate of 10^8^ s^-1^.

Based on the above discussion, the effects of strain rate on the interface activity of Ta/Cu multilayer systems have been studied in the literature. Meanwhile, it is not clear whether the strain rate plays an important role in the interface load-bearing and/or crystalline defect density, and thus, the mechanical properties of Ta/Cu nanocomposites. Additionally, the strain rate effects on the phase transition of these nanocomposites still remain unknown. To fill this research gap, the present work focuses on investigating the strain rate effects on the mechanical features, dislocation slip/twinning, and step-by-step phase transition during severe plastic deformation of pure Ta and Ta/Cu NCs under uniaxial tensile loading. To this end, first, a series of MD simulations are performed to examine the strain rate dependency of the mechanical properties of the aforementioned samples including yield stress, UTS, and flow stress. Then, the mechanisms underlying the plasticity induced in these samples under the simultaneous effect of Ta grain size and applied strain rate are investigated. In order to gain more insight into this issue, the microstructural evolution of all samples is thoroughly examined using various crystal structure analysis tools. In addition, since the strain rates applied in MD simulations are much higher than those in experimentally based studies, a new atomically informed Johnson–Cook (JC) constitutive model is developed to provide a practical way to predict the flow stress of polycrystalline nanomaterials at the strain rates comparable to the experimental ones.

## Methodology and simulation details

### Sample construction

In this study, two distinct samples have been constructed: one is used to represent pure Ta and the other is a Ta/Cu composite sample, which will be referred to as “Ta sample” and “Ta/Cu NC sample” in the following. It should be noted that to provide a better comparison, outside of the Cu inclusion, they are identical. All samples were built using the three-dimensional Voronoi tessellation technique^51^, which is employed to distribute randomly oriented grains at the specified average grain size. It is worth mentioning that in our previous studies^14,15^, we found a critical grain size of 8 nm as a breakdown of the Hall–Petch relationship for the introduced samples at 310 K.

**Figure 1:**
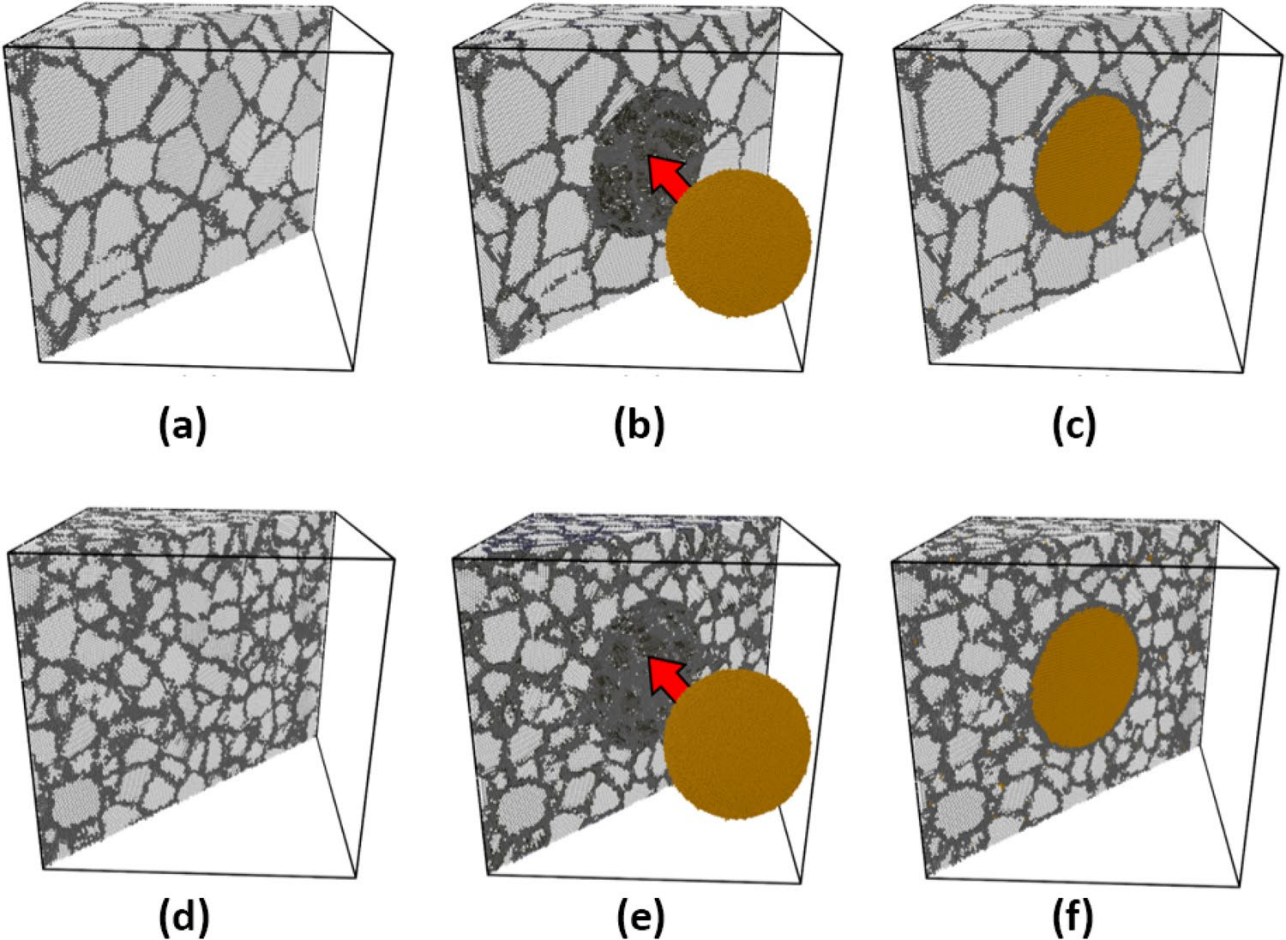
Construction steps of (**a**-**c**) coarse-grained and (**d**-**f**) fine-grained Ta/Cu NC samples. Brown color shows spherical Cu inclusion. Gray and dark gray colors indicate Ta matrix and Ta GB atoms, respectively. Taken from OVITO (version 2.9 27Jul2017 https://www.ovito.org).

Therefore, for both Ta and NC samples, average grain sizes of 4 and 8 nm were selected to represent the fine- and coarse-grained samples, respectively (see Fig. 1). In all simulations, a cubic representative volume element (RVE) with the dimensions of 30 × 30 × 30 nm^3^ was taken into account to avoid the effect of sample size on the reported data. Previous biological studies have shown that antibacterial properties of Ta-based implants can be considerably improved by the presence of Cu phase at an optimal loading of 5 wt.%^1^. Accordingly, a spherical copper NP with a diameter of 16.62 nm and an equivalent volume fraction of 8.9% was chosen to reach the mentioned weight percent of the Cu phase.

### Details of MD simulation

The open-source LAMMPS^52^ molecular dynamics simulator was employed to perform all simulations. The Angular-Dependent Potential (ADP) function developed by Pun et al.^53^ was used to model the Ta-Ta, Ta-Cu, and Cu-Cu interatomic interactions within the NC samples. Ab initio calculations on the lattice parameter and cohesive energy of Ta in BCC, FCC, and HCP crystalline structures have shown that this potential function can successfully analyze the phase stability of Ta compared to the well-known EAM potential function^49^. Consequently, using the ADP function could facilitate the characterization of phase transition in pure Ta and Ta/Cu NC samples subjected to severe plastic deformation at various strain rates. Initial velocities were sampled from a Maxwell–Boltzmann distribution at the body temperature (i.e., 310 K). Then, the velocity-Verlet algorithm with the time-step of 1 fs was utilized to integrate the equations of motion^54^. Moreover, periodic boundary conditions were assigned in all directions to obtain reliable properties using smaller RVEs. The Nosé–Hoover thermostat was implemented to control the temperature during all simulations.

Since initial samples are not energetically favorable, each simulation box was subjected to a multi-stage relaxation procedure before conducting the uniaxial tensile test. Targeting this purpose, first, to prevent atomic overlapping in the GB regions, the conjugate gradient (CG) algorithm was implemented to geometrically optimize the as-built samples. Then, to modify the morphology of GBs and Ta/Cu interface regions, after annealing at 310 K, the samples experienced further annealing stage. For a detailed description of the utilized relaxation process, the reader may refer to our recently published article^15^. It is worth mentioning that after multiple annealing stages, the flat morphology of GBs change to a curved one, producing microstructures similar to those of reported in the experimental studies^55^. Such an annealing protocol minimizes dislocation core energies of misfit dislocations located at the interfacial region^56^. During all annealing stages, the isothermal-isobaric barostat (NPT) was employed to ensure zero pressure in all three directions and a lack of residual thermal stress within the samples. After relaxation, to investigate the effect of strain rate on the mechanical properties and plastic deformation mechanisms of the introduced samples, the tensile test was applied along the opposite sides of the RVEs at the strain rates of 5 × 10^7^, 5 × 10^8^, 5 × 10^9^, and 5 × 10^10^ s^-1^. The elongation proceeded until the strain level of 50%. It should be noted that to ensure the application of uniaxial tension along the longitudinal direction, the transverse stress components were kept constant at zero to prohibit the lateral strains during the loading stage.

### Crystal structure analysis tools

In the present study, the OVITO software package was employed to analyze and visualize the microstructure of all samples at the atomic scale^57^. Also, changing the crystalline structure of the samples during the phase transition process was monitored using the Polyhedral Template Matching (PTM)^58^. This tool can accurately calculate the local crystal orientation of atoms in perfect crystalline regions, GBs, free surfaces, and interfaces. Accordingly, it can be a powerful tool for distinguishing these regions from each other. Dislocation density was obtained using the Dislocation Extraction Algorithm (DXA)^59^. We also utilized the Centro-Symmetry Parameter (CSP) analysis to identify the twinned and non-twinned regions^60^.

## Results and discussion

### Pure Ta and Ta/Cu NC samples under tension: on the role of strain rate

**Figure 2:**
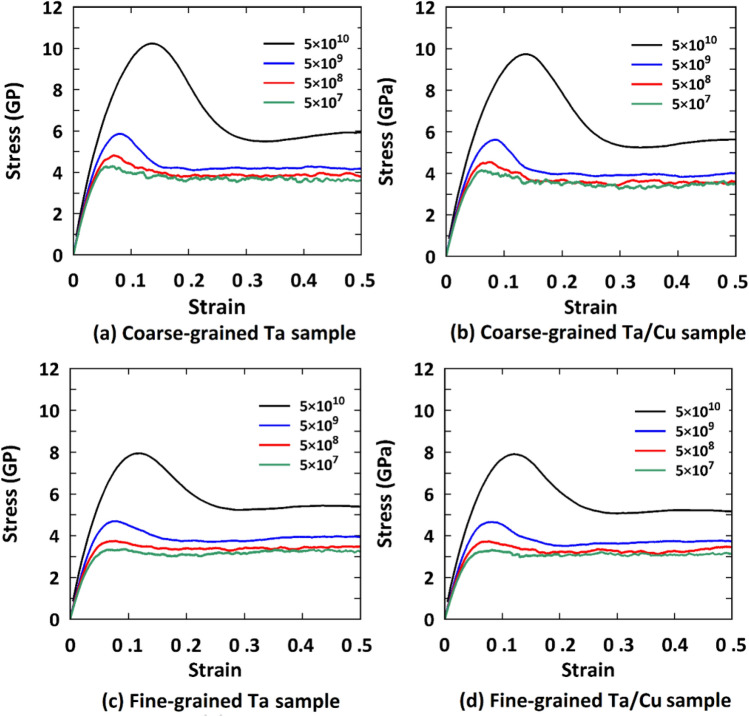
Strain rate dependent mechanical behavior of the pure Ta and Ta/Cu NC samples under tension. Legend gives strain rate in units of s^-1^.

Figure 2 displays the tensile stress–strain curves of the samples examined at various strain rates. It was generally revealed that decreasing the strain rate causes a reduction in their mechanical properties. This can be attributed to the fact that at high strain rate conditions, there is no appreciable chance of dislocation nucleation in polycrystalline structures^48,61^. Consequently, lower density of crystalline defects, especially dislocations, enhances their overall mechanical characteristics in these loading rates. To quantitatively analyze this issue, first, we study the initial slope of the stress–strain diagrams at small strain levels (i.e., ε < 2%). This “initial slope” corresponds to the Young’s modulus in the case of a purely elastic process; in our case of high strain rates, however, irreversible and hence plastic processes occur and lead to deviations of the initial slope from the elastic Young’s modulus. As the material undergoes strain, it needs to reach equilibrium at each time step if the changes are purely elastic and reversible. However, for large strain rates, this will not be possible any longer.

**Figure 3:**
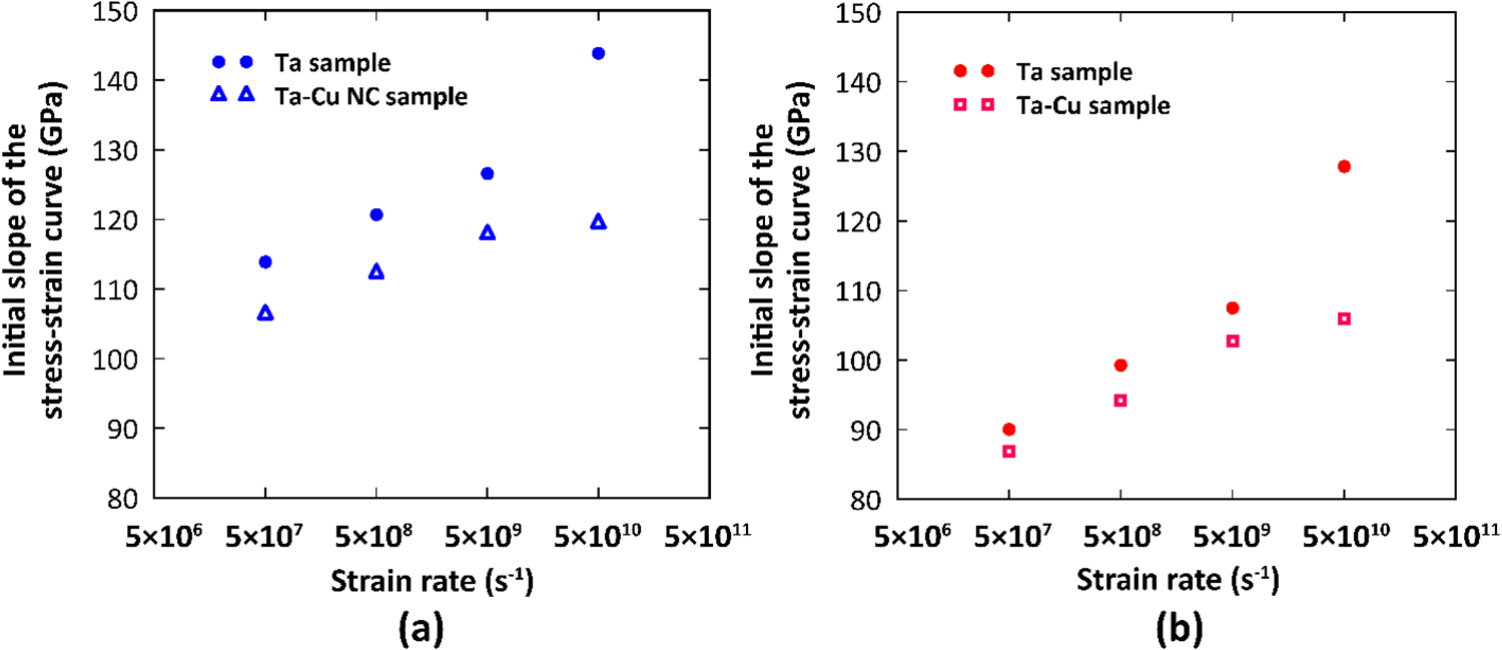
Strain rate dependent mechanical response of the pure Ta and Ta/Cu NC samples for small values of strain: (**a**) Coarse-grained samples, (**b**) Fine-grained samples.

As illustrated in Fig. 3, the presence of copper NP as a softer phase would lead to weakening of the Ta matrix. It was also found that at higher strain rates, the difference in the initial slope between the Ta and NC samples was even more apparent. In this context, Zhou et al.^33^ showed that Ta/Cu incoherent interfaces are more able to defect transferring at faster loading rates, resulting in a more pronounced role of the copper phase on the mechanical properties of Ta/Cu multilayered systems.

**Figure 4:**
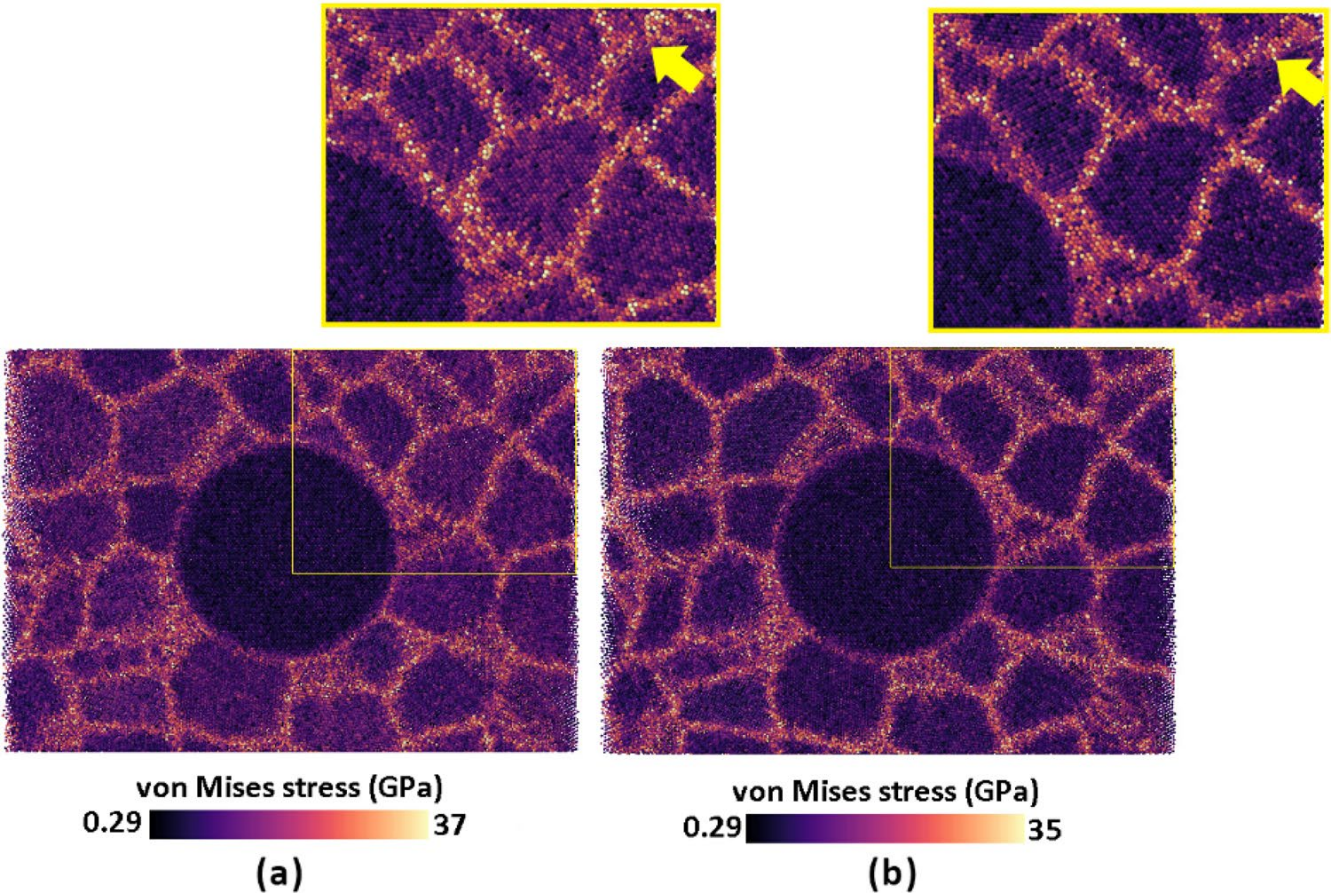
2% Offset stress distribution map by von Mises analysis for matrix grains of the coarse-grained NC sample at different strain rates: (**a**) 5 × 10^7^ s^-1^, (**a**) 5 × 10^10^ s^-1^. Yellow arrows indicate the highly stressed GB regions. Atoms are colored based on the von Mises analysis tool.

Recent studies on polycrystalline nanomaterials^62–64^ have found that the stress concentration in GB zones is higher than in grain interiors. This leads to some plastic deformation occurring in the GBs before the appearance of dislocations. Consequently, the change observed in the initial slope of the stress–strain curves can be attributed to the effect of GB plastic deformation. To gain a better understanding of the variations in the initial slope of the tested samples under different strain rates, we used the von Mises stress distribution to analyze the high-stress concentration regions in the coarse-grained Ta/Cu NC model. As disclosed in Fig. 4, GBs experience slightly higher stress at lower strain rates compared to their counterparts at higher rate conditions. This demonstrates that the reason behind the discussed increase in the initial slope of the mechanical behavior diagrams with strain rate is the irreversible plastic processes occurring within the models under low strain rate conditions, even at small values of imposed strain level. A similar behavior was also observed in other samples, as shown in Fig. 3.

**Figure 5:**
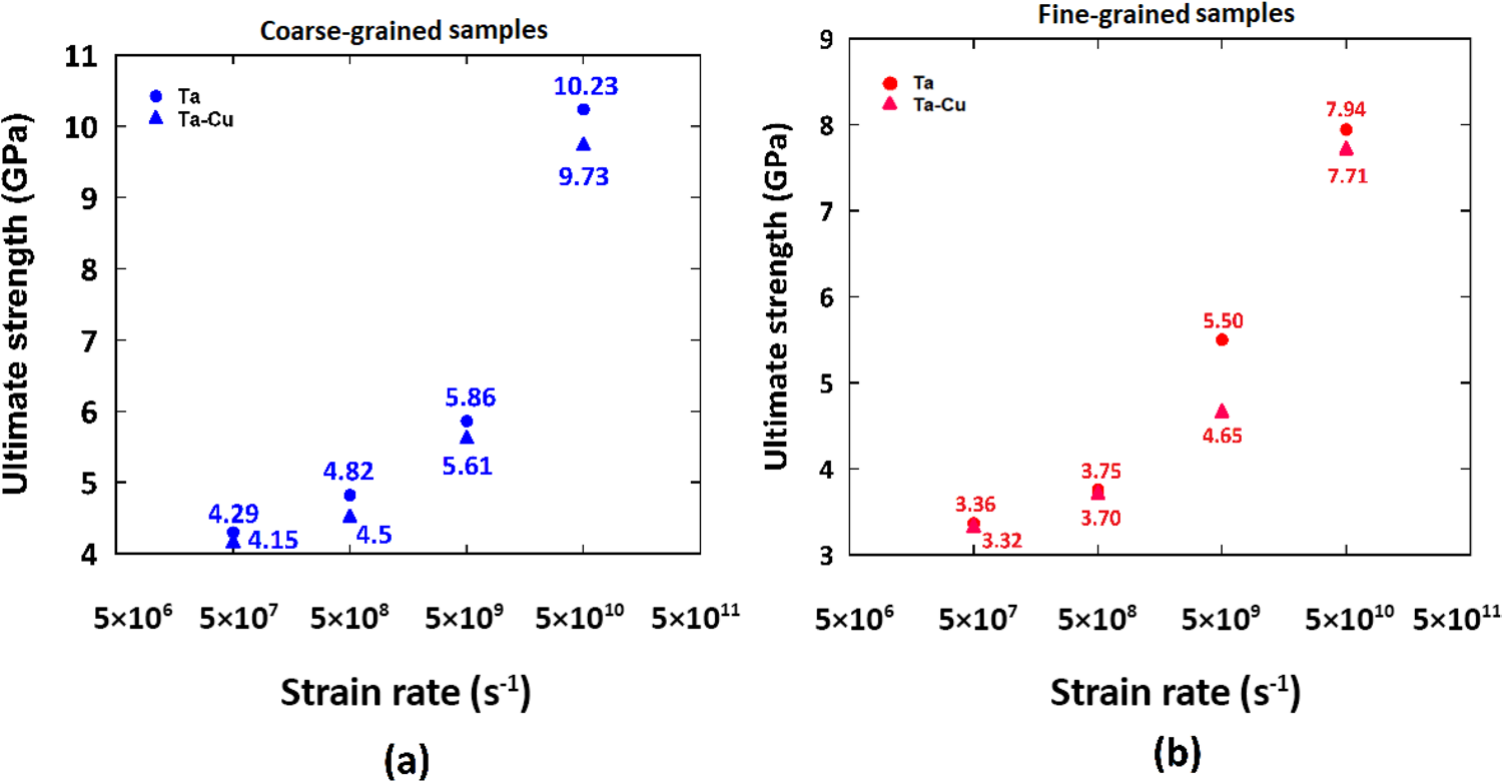
Comparison of the ultimate tensile strength of the pure Ta and Ta/Cu NC samples at various strain rates.

**Figure 6:**
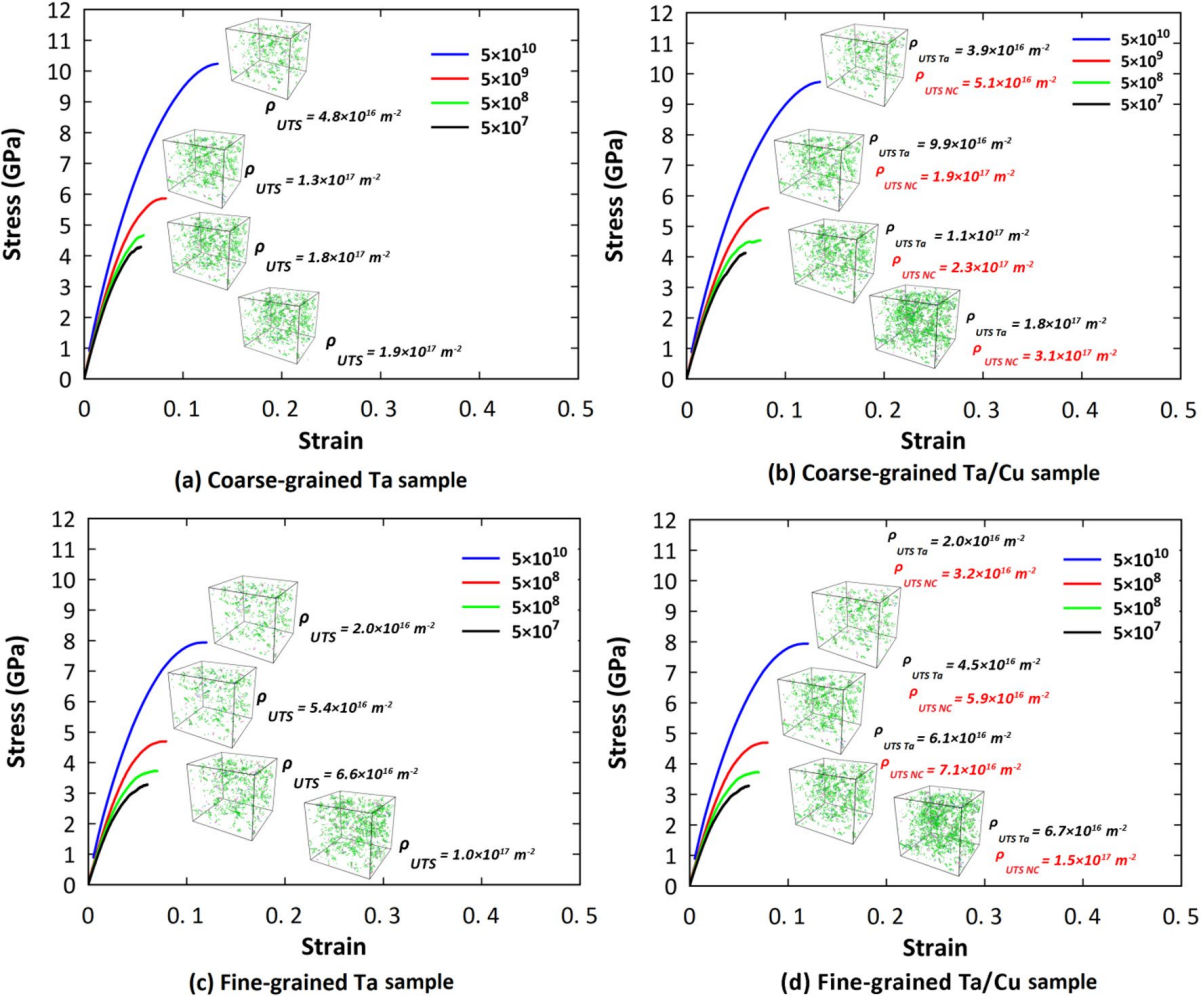
Dislocation density at the UTS point of the Ta and Ta/Cu NC samples. Legend gives strain rate in units of s^-1^. Configurations have been characterized by the DXA analysis provided by the OVITO program package (version 2.9 27Jul2017 https://www.ovito.org).

To further explore the effect of strain rate on the mechanical properties of the studied samples, we compared their UTS at various strain rates as illustrated in Fig. 5. It was deduced that the UTS values of the pure Ta and Ta/Cu NC samples are close to each other. However, the NC samples experience a slight reduction of the UTS compared to the pristine Ta. In this context, experimental observations by Rodriguez et al.^65^ showed that dislocation density and dislocation-twin interactions affect the UTS of materials. Similarly, an MD-based study by Hahn et al.^48^ demonstrated that dislocation slip and twinning determine the UTS of polycrystalline Ta in the strain rate interval of 10^7^ s^-1^ to 10^10^ s^-1^. Since grain boundary regions act as the main sources of dislocation nucleation, the contribution of dislocations to the UTS determination would be more pronounced by decreasing the average grain size of the Ta matrix to the values of 4 and 8 nm in our case studies. Accordingly, we performed a thorough analysis on the variation of dislocation density with the applied strain rate for the mentioned samples to understand the physics behind the UTS point changes. As disclosed in Fig. 6, the presence of the Cu phase would increase the dislocation density at the UTS point of the NC samples. This was attributed to the accelerated dislocation nucleation in nanocomposite systems resulting from the stress concentration at their interface region^15,66–68^. Meanwhile, as seen in this figure, the mentioned dislocation enhancement due to the addition of only 5 wt.% copper phase is not significant enough to cause a noticeable drop in the UTS point. These findings would manifest themselves in the results reported in Fig. 5, in which the UTS of the Ta and Ta/Cu NC samples seem quite similar.

Upon comparing the reported dislocation densities in Fig. 6b,d with those in Fig. 6a,c, a decrease in dislocation density can be observed for the matrix portion of the NC samples when compared to their pure Ta counterparts. This reduction can be attributed to the presence of the Cu phase located at the center of the Ta matrix, which leads to a decrease in GB density within the matrix. Furthermore, the plastic deformation of the Cu phase consumes energy that could otherwise be available for dislocation nucleation in the Ta matrix. Consequently, within the matrix part of the NC models, dislocations encounter fewer nucleation sites and limited nucleation energy, resulting in a reduction of the dislocation density values.

**Figure 7:**
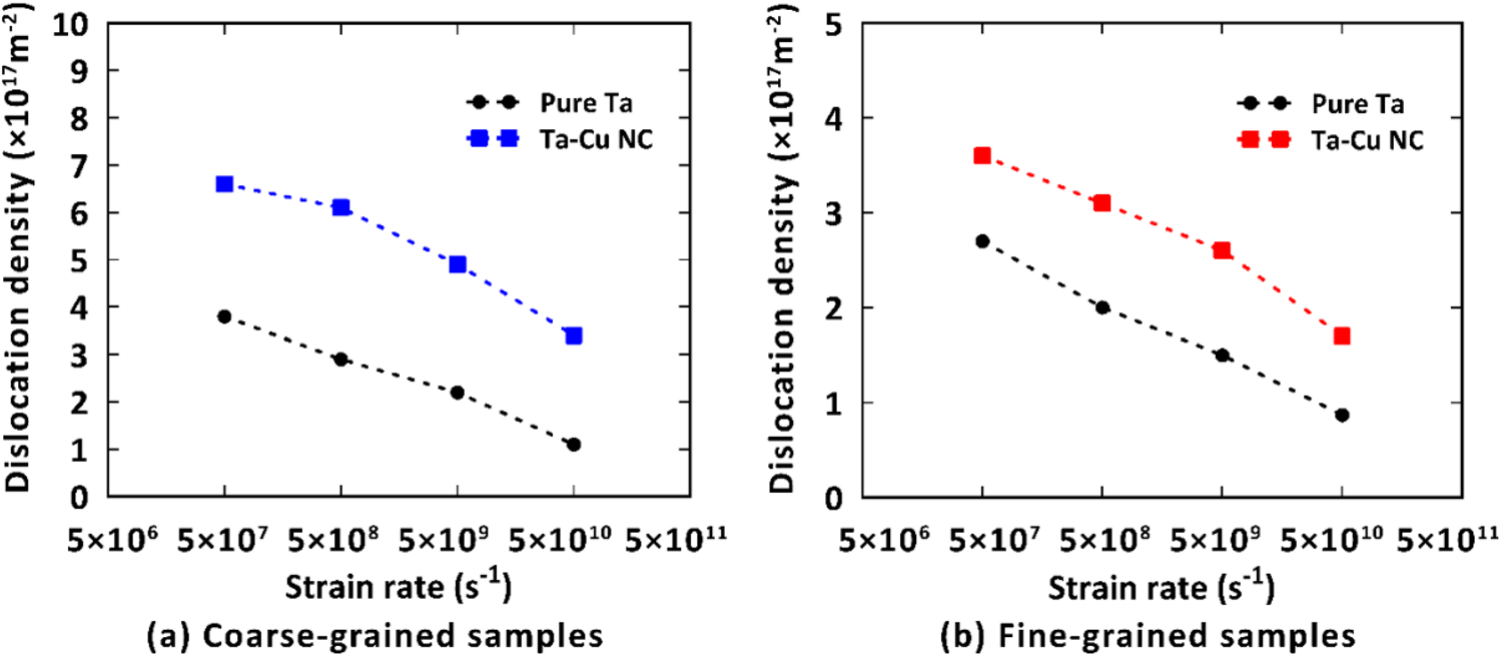
Dislocation density at the strain of 0.4 for the Ta and Ta/Cu NC samples. Configurations have been characterized by the DXA analysis provided by the OVITO program package (version 2.9 27Jul2017 https://www.ovito.org).

Flow stress is another important factor that is influenced by the strain rate. To shed further light on this issue, we also investigated the role of tension rate on the flow stress values for the samples we introduced. It is important to note that, in terms of flow stress fluctuations in the plastic zone, we considered the averaged stress values within the strain range of 0.3–0.5 as the flow stress. As shown in Fig. 2, decreasing the strain rate leads to a reduction in the flow stress of the mentioned samples. This indicates that dislocation-based activities are more effective in facilitating plastic deformation in these loading conditions. Therefore, we anticipate a strong correlation between flow stress values and dislocation density, similar to what has been discussed earlier for the UTS points. Figure 7 presents the dislocation density values at a strain of 0.4 (i.e., the middle of the mentioned strain range of 0.3–0.5), for the studied samples. As depicted in this figure, reducing the strain rate results in an increase in dislocation density across all samples, highlighting the significant role of strain rate in plastic flow, even at high strain levels. Furthermore, it can be observed that the presence of Cu NP leads to an enhancement in dislocation density, which subsequently reduces the required stress for initiating plastic deformation in the NC models. These findings are consistent with the results reported in Fig. 2, where the flow stress of the NC models exhibits lower values compared to their pure counterparts.

After examining the effect of strain rate on the mechanical properties of the studied samples, we further proceeded to determine the strain-rate-sensitivity (SRS) parameter of the NC samples as a key property for validation of the computational model. The SRS parameter ($m$) is defined as the slope of the log–log flow stress–strain rate curve^49^:

1 $$m = \left( {\frac{{\partial ln\sigma_{f} }}{{\partial ln\dot{\varepsilon }}}} \right).$$

**Figure 8:**
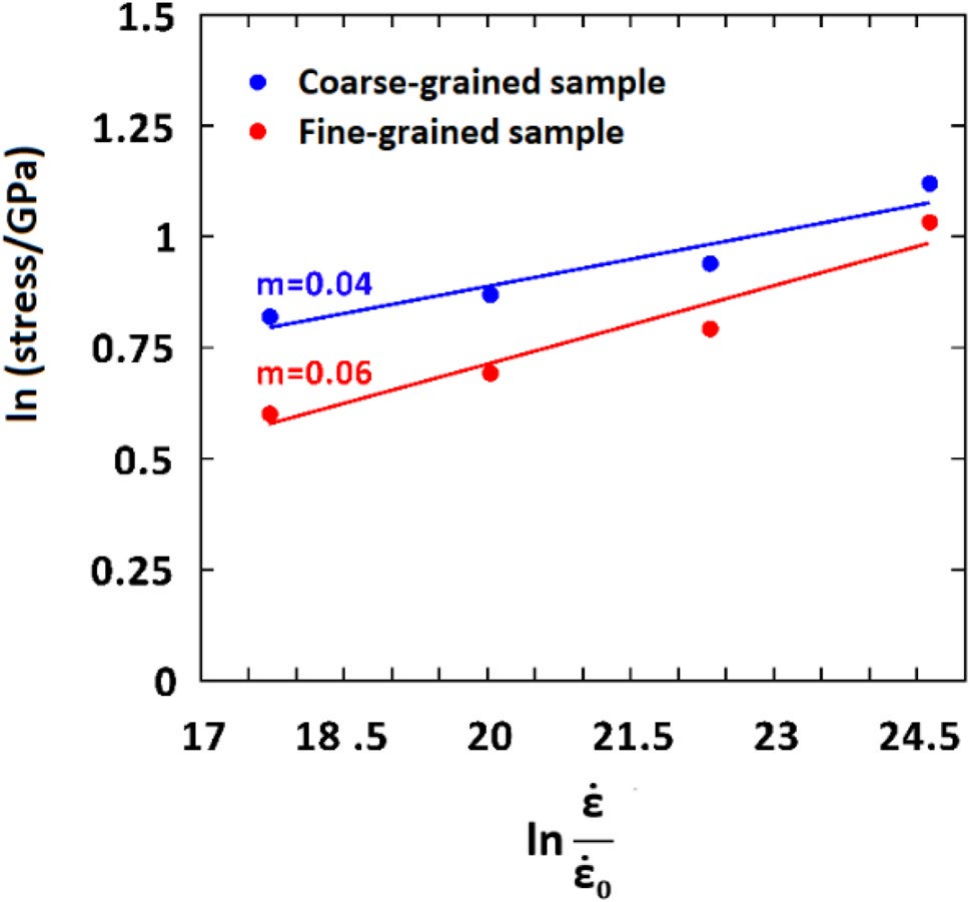
Log–log yield stress vs. strain rate curve for the coarse- and fine-grained pure Ta samples.

It has been demonstrated that SRS is a crucial factor in characterizing the strain rate effects on the plastic deformation mechanisms of metal-based nanomaterials. Having examined different types of logarithmic flow stress–strain rate curves, Sun et al.^69^ proposed that the yield stress (0.2% offset) can be a good choice for more accurate calculation of this factor. Employing this procedure, the SRS parameter was obtained as 0.04 for the coarse-grained Ta sample, which is equal to the reported experimental data for various coarse-grained bcc metals^70^ (see Fig. 8). Additionally, as disclosed in this figure, SRS increases from 0.04 to 0.06 with decreasing the grain size from 8 to 4 nm, showing a strong dependency of this parameter to the grain size. This issue is attributed to the activation of GB-mediated processes such as GB sliding, migration, and diffusion during the plastic deformation of fine-grained samples, as thoroughly discussed in the literature^71^. Similar correlation between the SRS parameter and the grain size has been reported experimentally for additively manufactured 316L stainless steels^72^. The mentioned GB-based activities have been observed in our fine-grained samples as will be shown later in “Low strain rate plastic deformation mechanisms: dislocation-based activities” section. Therefore, our findings are in line with the experimental observations and simulated data, confirming that the present samples and simulation method can precisely address the role of strain rate on the mechanical properties and deformation mechanisms of Ta-based polycrystalline nanomaterials. To ensure that the slight differences in the reported SRS values for the mentioned samples fall within the confidential intervals, we conducted 3 computations using various atomic initial velocities in each case. The standard error was subsequently determined to be 0.0012 and 0.0015 for the coarse- and fine-grained Ta models, respectively. This strengthens the validity of our comparison.

To further validate the simulation methodology, a *quantitative* comparison between the predicted values of flow stress and corresponding experimental data would be helpful. The selection was based on the fact that this mechanical property has demonstrated an important role on the plastic deformation of metallic nanomaterials^73^. Considering the fact that the examined strain rates in the present study are much higher than that of experimental-based observations, we used the well-known Johnson–Cook (JC) constitutive model for predicting the flow stress values at lower strain rates, which are inaccessible in MD simulation. This model can successfully predict the effect of temperature and strain rate on the von Mises flow stress as follows^74^:

2 $$\left. {{\upsigma }_{flow} = \left[ {A + B\varepsilon^{n} } \right]\left[ {1 + Cln\frac{{\dot{\varepsilon }}}{{\dot{\varepsilon }_{0} }}} \right][1 - \left( {\frac{{T - T_{R} }}{{T_{m} - T_{R} }}} \right)^{m} } \right].$$

In this equation, $\varepsilon$ is the equivalent plastic strain, and $\dot{\varepsilon }$ and $\dot{\varepsilon }_{0}$ are the applied and reference (i.e., 1 s^-1^) strain rates, respectively. Also, $T$ denotes the temperature of the experiment (i.e., 310 K), and $T_{R}$ and $T_{m}$ are the room temperature (298 K) and melting point (3301 K)^53^. Moreover, $A$, $B$, $n$, $C$, and $m$ are material constants. It is noted that the third bracket in Eq. (2) is related to the temperature effect, and thus, it would vanish at room temperature conditions. Substituting the mentioned constants for polycrystalline Ta reported in Table 1 and considering the value of 0.4 for $\varepsilon$, this equation can be simplified as:

3 $${\upsigma }_{flow} = \left( {362.15 {\text{MPa}}} \right) \left[ {1 + C ln\frac{{\dot{\varepsilon }}}{{\dot{\varepsilon }_{0} }}} \right].$$

**Table 1:** Experimental-based JC constitutive model constants for pure Ta^75^.

$A$	$B$	$n$	$m$
318.5 MPa	153.2 MPa	0.6	0.4

**Figure 9:**
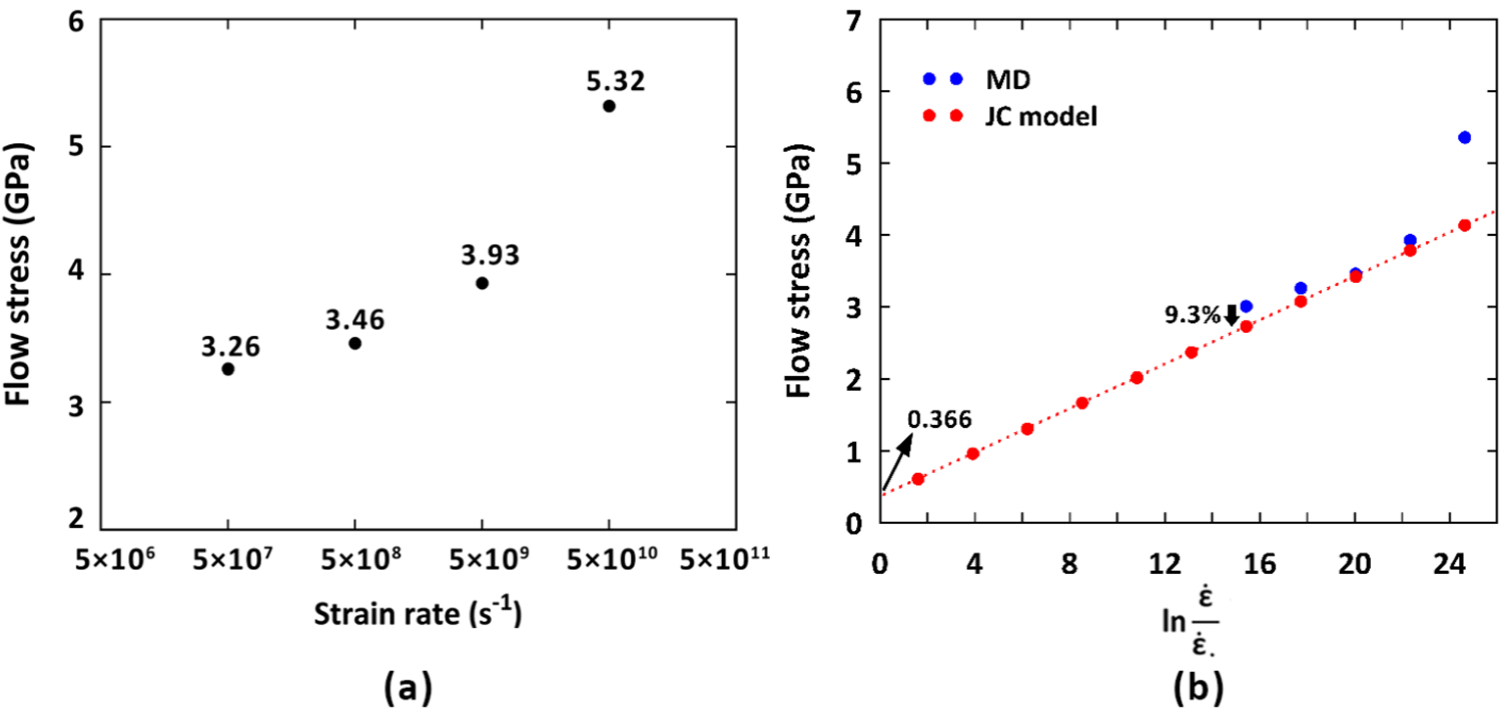
(**a**) Variation of flow stress with the applied strain rate for the fine-grained Ta sample, (**b**) JC predicted flow stress values for the fine-grained Ta sample at various strain rates.

As seen, a linear correlation was obtained between the flow stress and natural logarithm of strain rate from which, one could easily determine the only unknown $C$ parameter. Figure 9a discloses the flow stress values for the fine-grained pure Ta sample at various strain rates. Considering the data reported by MD simulations in Fig. 9b, it was found that the flow stress corresponding to the highest strain rate of 5 × 10^10^ s^-1^ is an outlier due to breaking down the linear trend of the variation of ${\upsigma }_{flow}$ with $ln\frac{{\dot{\varepsilon }}}{{\dot{\varepsilon }_{0} }}$. Therefore, it would be reasonable to ignore it in our computations. As such, having in hand the flow stress values at the strain rates of 5 × 10^7^, 5 × 10^8^, and 5 × 10^9^ s^-1^ in Fig. 9a, the mentioned $C$ parameter was calculated as 0.42.

It is noted that, in reality, at high strain rates, the system can experience heating due to energy dissipation through mechanisms such as plastic deformation or frictional effects. This deviation from constant temperature is a natural physical phenomenon that should be properly addressed in high strain rate loading conditions. To examine the dependency of our results to temperature variations, we have conducted a sensitivity analysis by examining available experimental data in the literature. As thoroughly discussed in^76^, an average temperature change of around 40 °C has been observed in Ti, Cu, and Al samples under severe strain rates with an assumed partial conversion of work into heat. So, considering the high melting temperature of nanocrystalline Ta models specified in^53^ as 3301 K, one can estimate that by replacing T with 350 K in Eq. (2), accounting for the reported temperature rises in previous experimental studies, the numerical coefficient in Eq. (3) would be changed a little, which validates the assumption of a constant temperature in our case studies.

To examine the accuracy of the results estimated by the proposed MD-based JC model, we compared the predicted flow stress at the newly examined strain rate of 5 × 10^6^ s^-1^ with the MD-obtained counterpart. As seen in Fig. 9b, the error percentage is below than 10%, showing that the developed JC model can be used for the determination of flow stress values at high strain rates. The same procedure would also be implemented for the coarse-grained sample leading to an error of 11.6% between the MD-based determined flow stress at the strain rate of 5 × 10^6^ s^-1^ with that of predicted by the developed JC model. According to Eq. (2), at the very low strain rate of 1 s^-1^, the second bracket of the model would be diminished, and the value of flow stress is primarily determined by the first and third terms. By substituting the corresponding parameter values from the experimental references introduced previously, the flow stress was calculated as 362.15 MPa. In line with predictions, this value is in good agreement with the experimental data reported for pure Ta at the same strain rate range (i.e., 350–900 MPa)^77,78^. As depicted in Fig. 9b, fitting a linear regression to the 3 data points obtained from MD simulations and extrapolating to the vertical axis results in a flow stress value of 366 MPa. This suggests that the provided data points from our simulations can effectively be utilized in the developed JC model to predict flow stress at low strain rates. It should be noted that there are no experimental studies on the samples having the same features such as grain size, strain rate, and temperature. However, comparing our outcomes with those of similar studies on the Ta samples show that the developed JC model could be *roughly* utilized to predict the flow stress values obtained in the experimental-based studies.

### Low strain rate plastic deformation mechanisms: dislocation-based activities

**Figure 10:**
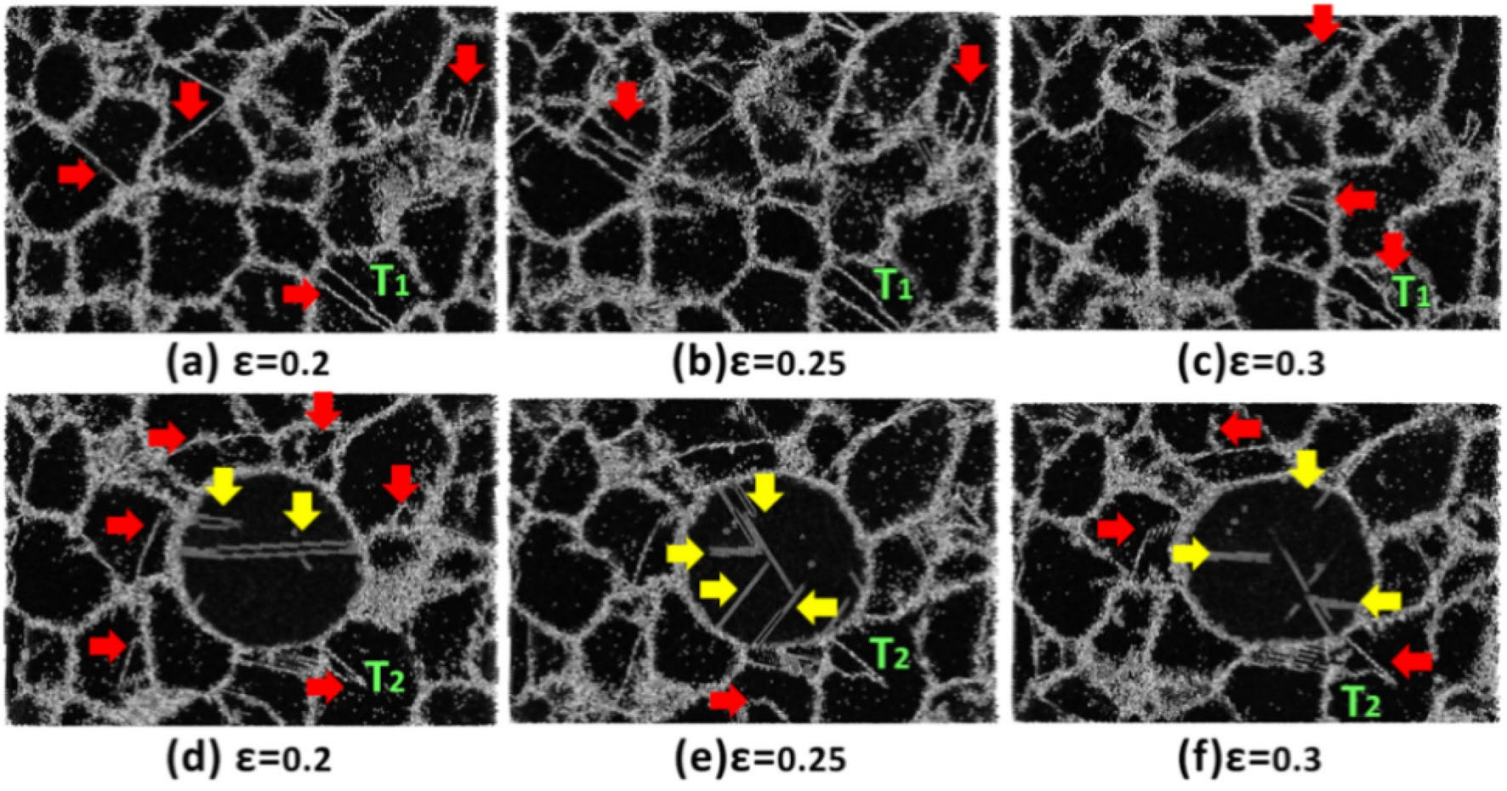
CSP analysis results for the coarse-grained samples at the lowest strain rate: (**a**–**c**) Pure Ta sample, (**d**-**f**) Ta/Cu NC sample. Red and yellow arrows show twins and stacking faults, respectively. Atoms are colored based on the CSP approach.

After examining the effect of strain rate on the mechanical properties of pure Ta and Ta/Cu NC samples, the next step involved exploring the underlying deformation mechanisms at various imposed strain rates. For this purpose, first, we probed the microstructural defects within the coarse-grained samples at the lowest strain rate (i.e., 5 × 10^7^ s^-1^). As thoroughly discussed in the literature, in these loading conditions, GBs and triple junction (TJ) points are the most important sites for twin formation at the initial stages of the induced plastic deformation in coarse-grained Ta samples. This was attributed to the development of highly localized stress fields in these regions^79–82^. To examine this issue for the present samples, we monitored the twin formation employing the CSP method (see Fig. 10). In this figure, red and yellow arrows indicate twins and SFs, respectively. As shown in Fig. 10a-c, the plastic deformation of pure Ta at low strain rate regimes is dominated by twin formation from GBs, which is followed by twin thickening with increasing the strain level. A similar trend can also be seen for the Ta/Cu NC sample as depicted in Fig. 10d-f. Comparing the twinning morphology in these two cases at the same strain levels (see T_1_ and T_2_ twins), one can notice the twin density reduction within the Ta matrix for the NC sample. This was ascribed to the interfacial load transfer in the NC sample, leading to the induced plasticity within the Cu NP. As disclosed by yellow arrows in Fig. 10d-f, the formation of SFs in the Cu phase provides sufficient evidence to demonstrate that twin thickening in the Ta matrix could be controlled by the plastic deformation of the Cu NP.

**Figure 11:**
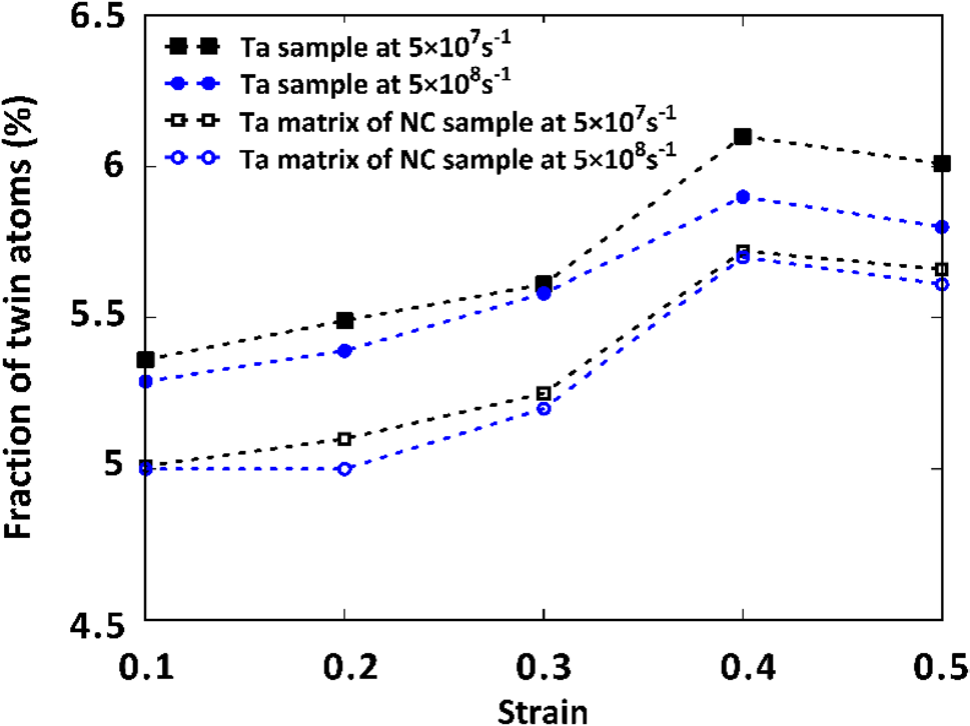
Quantitative analysis of the fraction of twin atoms in the coarse-grained models.

To provide *quantitative* evidence for these findings, we conducted a comparison of the fraction of twin atoms with respect to all atoms at various strains for the coarse-grained models analyzed in lower strain rate regimes. The results shown in Fig. 11 reveal a consistent decrease in the fraction of twin atoms when Cu NP is incorporated into the Ta matrix. This behavior can be attributed to the load transfer occurring at the interfacial area of the NC sample, which leads to the transfer of applied stress from the matrix to the copper NP, as strongly supported by the data presented in Fig. 10. Moreover, as illustrated in Fig. 11, the twin density of the samples exhibits a slight decrease as the strain rate increases. This phenomenon arises due to the limited time available for twin nucleation under these loading conditions, resulting in a lower density of crystalline defects. Consequently, this reduction in twin density contributes to the overall enhancement of the mechanical properties of the samples at higher strain rates.

**Figure 12:**
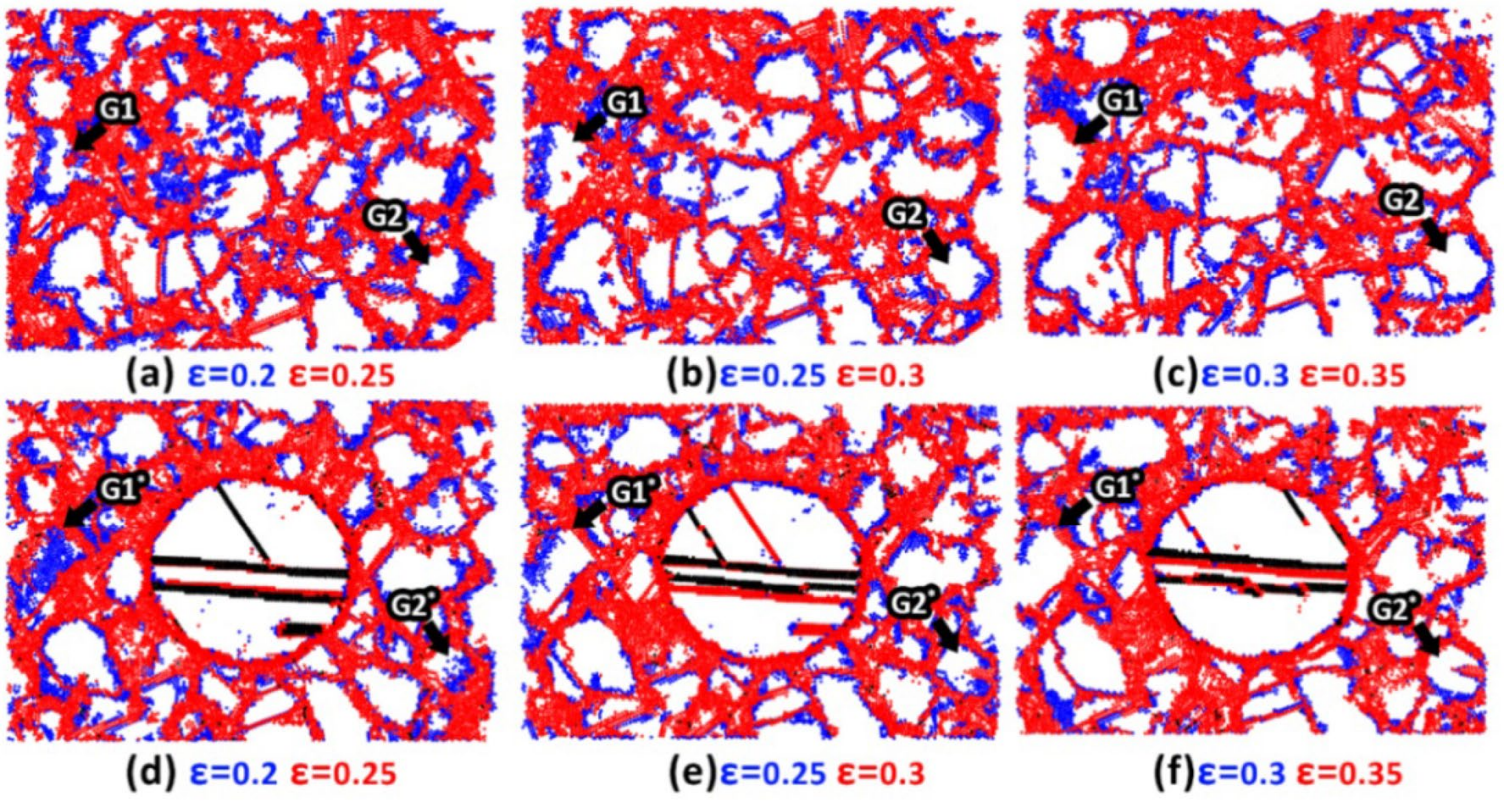
Evaluation of GBs of the fine-grained samples subjected to the lowest tensile strain rate: (**a**–**c**) Pure Ta sample, (**d**-**f**) Ta/Cu NC sample. Blue and red maps update the GB locations at various strain levels. Black arrows show the location of G_1_ and G_2_ grains in the pure Ta sample and their corresponding grains in the NC sample labled by G_1_^*^ and G_2_^*^. Images have been created employing the OVITO program package (version 2.9 27Jul2017 https://www.ovito.org).

It has been demonstrated that at low strain rates, passing the critical grain size, twins are no longer responsible for the plasticity induced within the polycrystalline materials and their plastic deformation is mainly under the influence of GB-based activities. In our previous work^15^, this issue has been thoroughly addressed for the ultra-fine-grained polycrystalline Ta samples. It was revealed that for the Ta samples with grain sizes smaller than 8 nm, GB-based phenomena such as sliding, rotation, and migration govern their plastic deformation. Here, to get more insight into the mechanisms governing the plastic deformation of fine-grained NC samples at the mentioned lowest strain rate, employing the PTM analysis, we compared the location of GB atoms at various strain levels for Ta and Ta/Cu NC samples with an average grain size of 4 nm. These regions have been colored by blue and red maps at the strains of 0.2 and 0.25 for Ta (Fig. 12a) and NC (Fig. 12d) samples, respectively. Additionally, to better probe the GB dynamics, the mentioned maps have been updated for the other strain intervals as depicted in Fig. 12. It was found that GB migration and grain elongation occur during the deformation of both samples. However, GB atoms are more easily displaced in the pure Ta case. For a visual representation of the subject, the reader may refer to Fig. 12 to compare the GB location of grain G2 in the Ta sample with that of its corresponding grain (i.e., G2*) of the NC sample at each strain level. Similar to the coarse-grained NC case, this was attributed to the formation of twins and SFs within the softer Cu phase as shown by red and black atoms in Fig. 12d-f.

### High strain rate plastic deformation mechanisms: step-by-step phase transition

Moving on to the next item, we proceeded to explore the mechanisms dominating the plastic deformation of the studied samples at higher values of strain rate. Experimental data have shown that stable α-tantalum (BCC) structure can change to β-tantalum (tetragonal), ω-tantalum (HCP), and FCC-structured Ta under severe tensile loading conditions^83,84^. However, the mentioned phase transition is a reversible phenomenon due to the instability of the FCC and HCP structures of Ta. Consequently, this metal is always seen in the α-phase. In this context, utilizing HRTEM, Janish et al.^38^ distinguished FCC and BCC phases in ultra-fine-grained Ta samples. Carrying out MD simulations, Li et al.^50^ successfully detected BCC-to-FCC phase transition in Ta single crystals subjected to high tensile strain rates. It has been well established that in single crystalline metals, solid–solid phase transition occurs through breaking atomic bonds by the shear stress components of the applied tensile stress^38,85^. Despite these efforts, there is a gap in the open literature regarding the phase transition in polycrystalline structures at high strain rate loading conditions.

**Figure 13:**
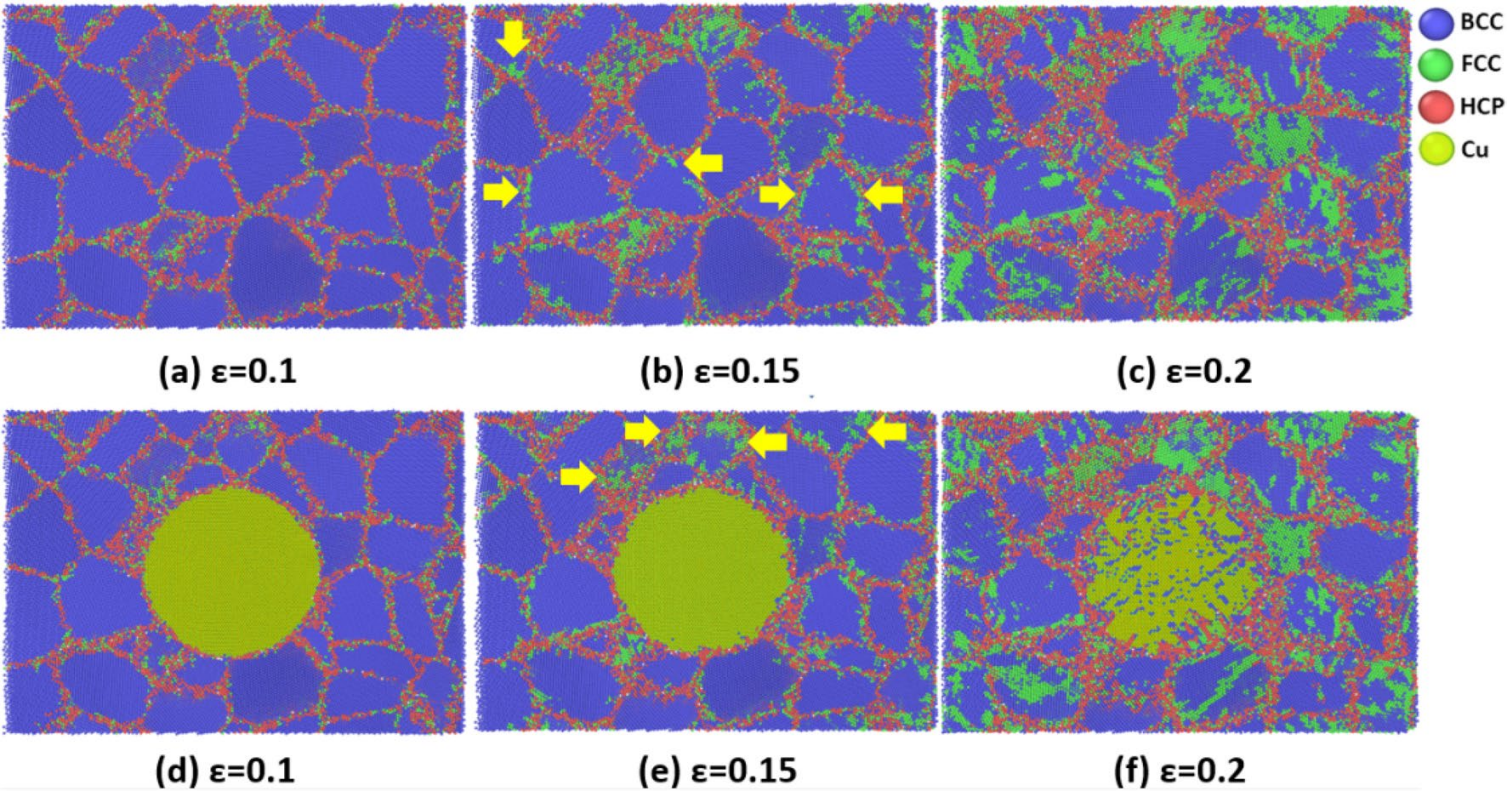
Step-by-step BCC-to-FCC phase transition in the coarse-gained samples: (**a**–**c**) Pure Ta sample, (**d**–**f**) Ta/Cu NC sample. Yellow arrows indicate phase transition initiation sites.

**Figure 14:**
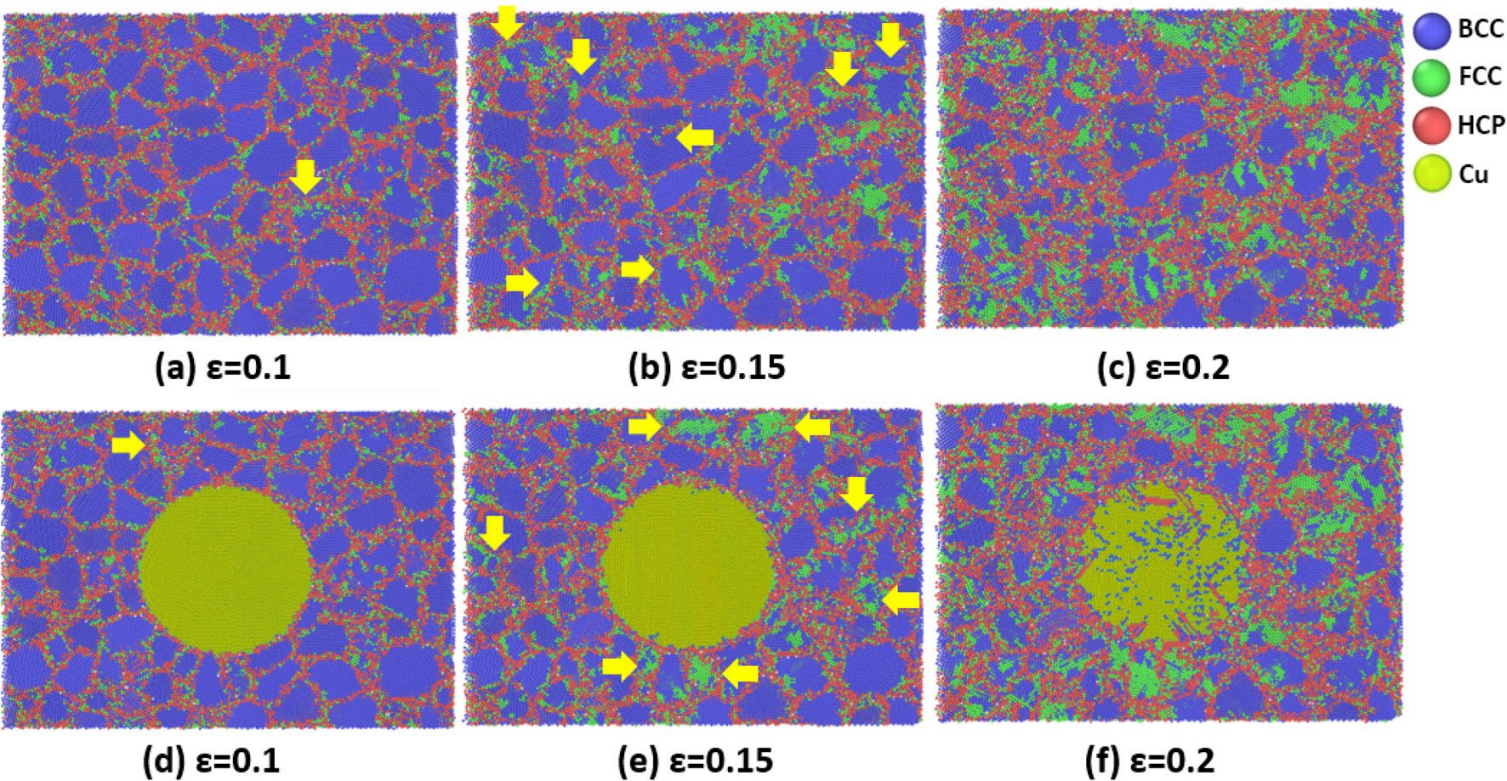
Step-by-step BCC-to-FCC phase transition in the fine-gained samples: (**a**–**c**) Pure Ta sample, (**d**–**f**) Ta/Cu NC sample. Yellow arrows indicate phase transition initiation sites.

To provide a more complete picture of how the discussed phase-transition can be affected by the GB-based activities, we took a closer look at the microstructural changes of the studied coarse-grained samples at the highest strain rate of 5 × 10^10^ s^-1^. As illustrated by yellow arrows in Fig. 13, phase transition expands from TJ points and GB zones to the grain interiors in some grains of both Ta and Ta/Cu NC samples. Therefore, it was concluded that phase transition is strongly dependent on the GB density in polycrystalline materials. Similar to the coarse-grained samples, GBs transfer shear stress components to the grain interiors of the fine-grained samples, resulting in their phase transition (see Fig. 14). It is worth noting that compared to their coarse-grained counterparts, more grains start the phase transition from GBs in these samples. However, the fraction of fully FCC-structured grains is lower in the finer grain-sized cases. It arises because in these samples, the imposed load would be distributed on the higher density of GBs, which leads to the reduction of the shearing force contribution for each grain.

**Figure 15:**
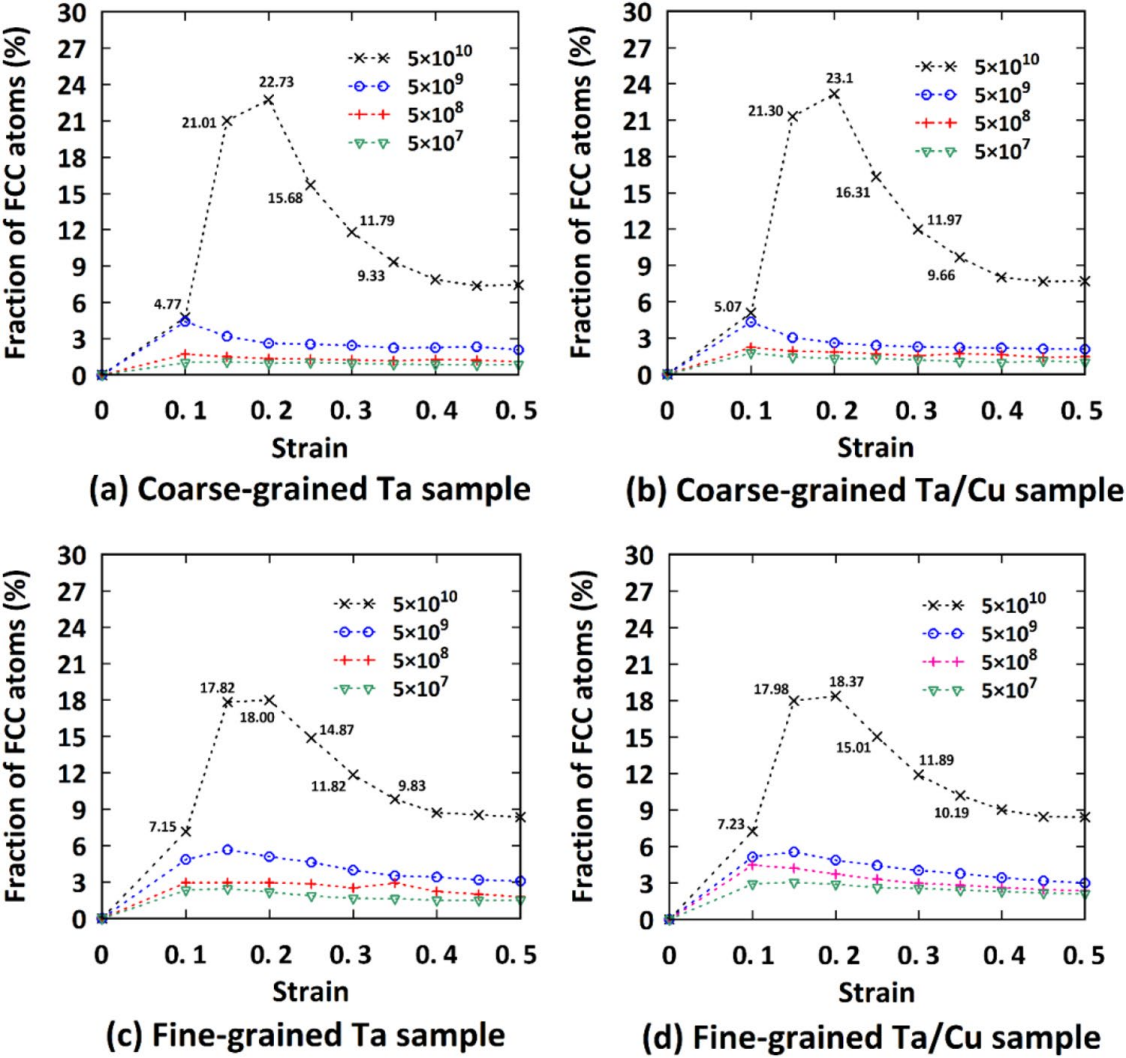
Quantitative analysis of the strain rate dependent microstructural changes of the introduced samples at various strain levels. Legend gives strain rate in units of s^-1^.

To complete the discussion, using the CNA tool, a quantitative analysis has also been provided in Fig. 15 to determine the fraction of FCC atoms in all pure Ta and NC samples at various strain rates. As mentioned earlier, a high-stress level is needed to break atomic bonds during the phase transition process. Therefore, as we expected, only at the highest strain rate, such FCC structures can be observed, supporting the fact that in these severe tensile loading conditions, BCC-to-FCC phase transition governs the plastic deformation of polycrystalline metals and their composites. Additionally, as previously discussed, the fraction of FCC atoms in fine-grained samples is lower than that of coarse-grained samples, which supports the mentioned relationship between the phase transition and density of GBs. Moreover, it is evident that with increasing the strain level, the FCC structure is changed back to the BCC one confirming the instability of this phase in the Ta-based materials. These findings are in good agreement with the data reported by Li et al.^50^ in the case of single crystalline Ta sample.

## Conclusion

In this paper, the effect of strain rate on the mechanical properties and plastic deformation mechanisms of coarse- and fine-grained pure Ta and Ta/Cu NCs was investigated by MD simulations at various tensile strain rates. For this purpose, first, the strain rate-dependent mechanical properties of the introduced samples were thoroughly examined. It was found that decreasing the applied strain rate would cause a significant reduction in the initial slope of the stress–strain curve, yield strength, and ultimate tensile strength of the samples. An increase in dislocation density was identified as the main reason for the observed decreasing trend at lower strain rate conditions. In addition, it was inferred that the presence of Cu NPs would reduce the mechanical features of the Ta matrix by promoting the dislocation nucleation in the interfacial region. The comparison of the strain-rate-sensitivity parameter of the studied samples with those reported experimentally in the literature demonstrated that the implemented simulation methodology and its details could be reliable for characterizing the mechanical behavior and plastic deformation mechanisms of nanocrystalline Ta-based materials.

To further examine this issue, a newly developed JC model was proposed based on the archived MD data to predict the flow stress of the samples at the strain rates similar to those used in experimentally based studies. After verifying the computational model, the microstructural evolution of all samples was examined using various crystal structure analysis tools to investigate the effect of strain rate on the governing deformation mechanisms.

Depending on the strain rate and Ta grain size, the plastic deformation mechanisms could be divided into three main categories, namely: dislocation-based activities, GB-based phenomena, and step-by-step phase transition. It was shown that at lower strain rates, dislocation slip and twinning are responsible for the deformation of coarse-grained samples. However, under these loading conditions, plasticity in the fine-grained samples is induced by the grain boundary activities such as GB sliding, migration, and grain elongation. The PTM analysis results showed that only at the highest strain rate of 5 × 10^10^ s^-1^, the BCC-to-FCC phase transition initiated from the GBs controls the plastic deformation of all samples. The present findings could give a deeper insight for the design of Ta/Cu therapeutic implants.

## Data Availability

All data used for this study are contained in this article.
